# Differential Cytotoxic Potential of Silver Nanoparticles in Human Ovarian Cancer Cells and Ovarian Cancer Stem Cells

**DOI:** 10.3390/ijms17122077

**Published:** 2016-12-12

**Authors:** Yun-Jung Choi, Jung-Hyun Park, Jae Woong Han, Eunsu Kim, Oh Jae-Wook, Seung Yoon Lee, Jin-Hoi Kim, Sangiliyandi Gurunathan

**Affiliations:** 1 Department of Stem Cell and Regenerative Biotechnology, Konkuk University, Seoul 143-701, Korea; yunjungc@konkuk.ac.kr (Y.-J.C.); saladinhyun@naver.com (J.-H.P.); woong1211@naver.com (J.W.H.); np-gennao@hanmail.net (E.K.); ohjw62@gmail.com (O.J.-W.); jhkim541@konkuk.ac.kr (J.-H.K.); 2 Swine Consulting Group, HanByol Farm Tech, Gyeonggi 463-785, Korea; kissleevet@gmail.com

**Keywords:** silver nanoparticles, ovarian cancer cells, ovarian cancer stem cells, cytotoxicity, cell viability, cancer therapy

## Abstract

The cancer stem cell (CSC) hypothesis postulates that cancer cells are composed of hierarchically-organized subpopulations of cells with distinct phenotypes and tumorigenic capacities. As a result, CSCs have been suggested as a source of disease recurrence. Recently, silver nanoparticles (AgNPs) have been used as antimicrobial, disinfectant, and antitumor agents. However, there is no study reporting the effects of AgNPs on ovarian cancer stem cells (OvCSCs). In this study, we investigated the cytotoxic effects of AgNPs and their mechanism of causing cell death in A2780 (human ovarian cancer cells) and OvCSCs derived from A2780. In order to examine these effects, OvCSCs were isolated and characterized using positive CSC markers including aldehyde dehydrogenase (ALDH) and CD133 by fluorescence-activated cell sorting (FACS). The anticancer properties of the AgNPs were evaluated by assessing cell viability, leakage of lactate dehydrogenase (LDH), reactive oxygen species (ROS), and mitochondrial membrane potential (mt-MP). The inhibitory effect of AgNPs on the growth of ovarian cancer cells and OvCSCs was evaluated using a clonogenic assay. Following 1–2 weeks of incubation with the AgNPs, the numbers of A2780 (bulk cells) and ALDH^+^/CD133^+^ colonies were significantly reduced. The expression of apoptotic and anti-apoptotic genes was measured by real-time quantitative reverse transcriptase polymerase chain reaction (qRT-PCR). Our observations showed that treatment with AgNPs resulted in severe cytotoxicity in both ovarian cancer cells and OvCSCs. In particular, AgNPs showed significant cytotoxic potential in ALDH^+^/CD133^+^ subpopulations of cells compared with other subpopulation of cells and also human ovarian cancer cells (bulk cells). These findings suggest that AgNPs can be utilized in the development of novel nanotherapeutic molecules for the treatment of ovarian cancers by specific targeting of the ALDH^+^/CD133^+^ subpopulation of cells.

## 1. Introduction

Ovarian cancer is the fifth most common cancer among all types of cancer, and the second most common gynecological malignancy. According to the American Cancer Society (ACS), 22,280 women will receive a new diagnosis of ovarian cancer, and 14,240 women will die from ovarian cancer in 2016 [1]. Most cases are diagnosed in the advanced stage [2]. The preliminary treatment was performed such as surgery is followed by platinum-based chemotherapy in women with ovarian cancer [3,4]. Although most women respond to primary treatment, eventually there is chemoresistance. The recurrence of cancer is due to a high degree of heterogeneity within ovarian tumors, a key feature of ovarian cancer, and between different ovarian cancer subtypes. In addition, a paucity of widely expressed therapeutically targetable genetic changes restricts effective treatment options [5]. Combination of chemotherapy is initially beneficial for ovarian cancer patients but eventually resistance develops [6]. In addition, ovarian cancer cells are a heterogeneous population of cells, with increased tumorigenicity and differentiating capacity compared with other cancer stem cells (CSCs) [7]. CSCs were isolated from various cancer cells based on either differential expression of cell surface markers or differential biochemical properties [8,9,10]. Aldehyde dehydrogenase (ALDH) has been proposed together with CD133 to identify the CSC population in hepatocellular carcinoma [11] and ALDH^+^ cells are seems to be capable of directly generating tumors in vivo [10]. Among different subpopulations of CSCs, ALDH^+^ and CD133^+^ populations of cells were able to form three-dimensional spheres more efficiently than their negative counterparts. Further, ALDH^+^, CD133^+^, and ALDH^+^/CD133^+^ cells are capable to form tumors rapidly [9]. Choi et al. [12] reported that ALDH^+^/CD133^+^ subpopulations of cells could generate all four type of ALDH^+/−^CD133^+/−^ cell populations and had a clear branched differentiation hierarchy. Therefore, targeting CSCs is a vital aspect of cancer therapy.

Increasing evidence suggests that CSCs contribute to acquiring chemotherapy resistance across a broad range of malignancies, and a better understanding of CSCs could aid in the design of new therapies that improve the efficacy of chemotherapy [13]. CSCs are capable of unlimited self-renewal, which would give rise to tumorigenicity, and drug resistance for long term [14,15]. CSCs are able to grow and spread to maintain tumorigenic potential [12]. The potential of the CSCs population in ovarian cancer cells is defined by cell markers, including ALDH enzymatic activity and the stem cell marker CD133 [16,17], suggesting the potential role of ALDH^+^/CD133^+^ cells as the ovarian cancer cells of origin [18]. The mechanism of chemoresistance of cancer stem cells is a result of several factors including enhanced ALDH activity, ATP-binding cassette transporters (ABC) transporter expression, B-cell lymphoma-2 (BCL2)-related chemoresistance, enhanced DNA damage response, and activation of key signaling pathways [19].

Nanoparticles have become widely utilized because of their unique properties and diverse applications in industry, cosmetics, biotechnology, and nanomedicine. Silver nanoparticles (AgNPs) are one of the most commercialized nanoparticles worldwide among various nanomaterials. AgNPs are used as antibacterial, anticancer, and antiangiogenic agents because of their unique properties, such as optic and catalytic features, and they have potential for use in the creation of novel and advanced functional materials [20,21,22]. Therefore, the unique toxicity profiles of AgNPs may also offer an opportunity to exploit specific vulnerabilities in cancer, provided that an appropriate disease target could be identified (likely CSCs). The potential therapeutic efficiency of any anticancer drug is based on targeting specific cells; particularly, distinguishing between cancer cells and normal cells based on differential sensitivities of the two cell type [23]. The significant challenges in the treatment of ovarian cancer are due to multiple ovarian histophenotypes, various possible sites of disease origin, and differential hierarchal contributions of multiple CSC populations [17]. Therefore, the identification, functional characterization, and therapeutic targeting of ovarian CSCs are necessary. To the best of our knowledge, there is no study on the cytotoxic effect of AgNPs on different subpopulations of ovarian CSCs. Therefore, we designed a study based on the following objectives: the first objective of this study was to isolate different subpopulations of CSCs from human ovarian cancer cells using different surface markers such as ALDH^+^/CD133^+^. The second objective of this study was to evaluate the cytotoxic potential of AgNPs on bulk cancer cells (A2780) and different subpopulations of ovarian cancer stem cells (OvCSCs). The third objective was to assess the effect of AgNPs on OvCSC self-renewal capacity, using the colony formation assay, and to elucidate the mechanisms of apoptosis induced by AgNPs in bulk cancer cells (A2780) and a specific subpopulation of OvCSCs, ALDH^+^/CD133^+^ cells.

## 2. Results and Discussion

### 2.1. Characterization of Silver Nanoparticles (AgNPs)

**Figure 1:**
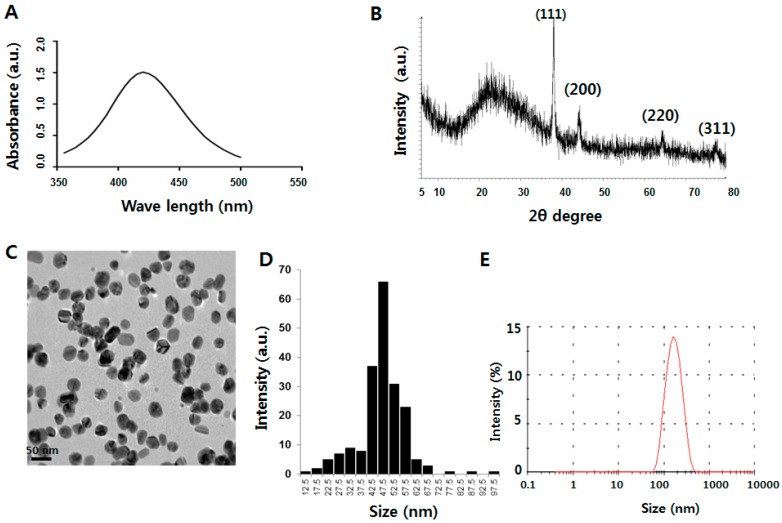
Characterization of silver nanoparticles (AgNPs) using various analytical techniques. (**A**) The absorption spectrum of AgNPs exhibited a strong broad peak at 420 nm and observation of such a band is assigned to surface plasmon resonance of the particles (**A**); X-ray diffraction (XRD) pattern of silver nanoparticles (**B**); TEM images of AgNPs (**C**); particle size distributions from transmission electron microscopy images (**D**); Several fields were photographed and used to determine the diameter of AgNPs, the average range of observed diameter was 47.5 nm. Size distribution analysis of AgNPs using dynamic light scattering (DLS) (**E**).

The aim of this experiment was to understand the anticancer effect of AgNPs in ovarian cancer cells and OvCSCs. Characterization of AgNPs was performed according to methods previously described [24]. First, we performed preliminary characterization using UV-VIS spectroscopy (Mecasys Co., Seoul, Korea). he UV-VIS absorption spectra were measured, and peaks was observed in the range of 350–550 nm, with a strong peak located at 420 nm (Figure 1A), which is typical characteristic feature of AgNPs. To confirm the crystalline nature of the particles, the X-ray diffraction (XRD) pattern of the AgNPs was evaluated; it is shown in Figure 1B. The XRD results clearly showed that the AgNPs were crystalline in nature, and four prominent peaks were observed. Transmission electron microscopy (TEM) is a valuable tool for analysis of the surface morphology and shape of nanoparticles. As shown in Figure 1C, the diameter and morphology of AgNPs were analyzed by TEM. The TEM image shows well-dispersed, uniform, spherical-shaped particles. We measured the particle size distributions from transmission electron microscopy images from more than 200 particles, and the distribution is presented. The average range of observed particle diameter was 47.5 nm (Figure 1D). Although the average size was 47.5 nm, the AgNP colloidal suspension contained differently sized particles with a diameter range mostly between 42 nm and 57 nm. The size distribution was further confirmed by dynamic light scattering (DLS), which is used to evaluate particle size and size distribution of nanomaterials in solution [21]. DLS analysis shows that AgNPs had an average size of 50 nm (Figure 1E). However, the range of sizes was higher than that obtained using TEM because of Brownian motion. 

### 2.2. AgNPs Induce Dose- and Time-Dependent Effects on Cell Viability in Human Ovarian Cancer Cells

**Figure 2:**
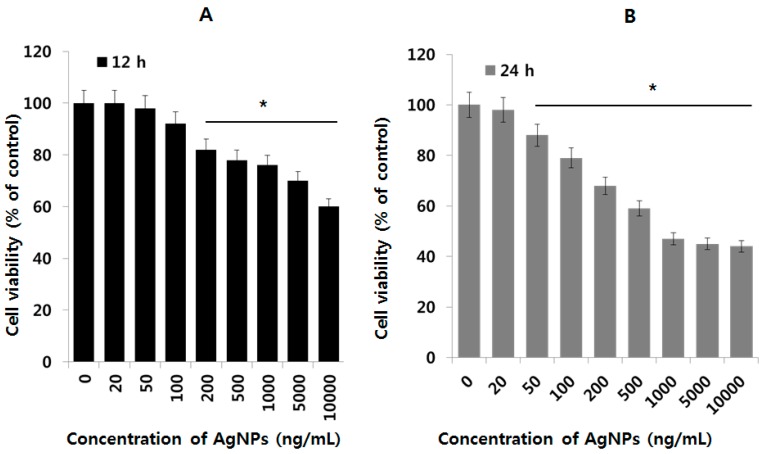
Dose and time dependent effect of AgNPs on cell viability of human ovarian cancer cells. The viability of A2780 human ovarian cancer cells was determined after 12 h exposure to different concentrations of AgNPs using the CCK-8 assay (**A**); the viability of A2780 human ovarian cancer cells was determined after 24 h exposure to different concentrations of AgNPs using the CCK-8 assay (**B**); the results are expressed as the mean ± standard deviation of three independent experiments. The viability of treated cells compared to the untreated cells was analyzed using the Student’s *t*-test (* *p* < 0.05).

Before examining the effect of AgNPs on OvCSCs, we first examined the cytotoxic effects of AgNPs on A2780 cells (bulk) using a cell viability assay. A2780 cells are parental cells, which are used for isolation of OvCSCs. To determine the effect of AgNPs on A2780 cells, A2780 cells were exposed to different concentrations of AgNPs ranging from 20 ng/mL to 10,000 ng/mL for 12 and 24 h, and then cell viability was assessed using the cell counting kit (CCK-8) assay (Figure 2). The results of the CCK-8 assay, which measured water-soluble formazan dye produced by metabolic activity of live cells, showed that cell viability was decreased after exposure to AgNPs in a time- and dose-dependent manner. A2780 cells were treated with various concentrations of AgNPs for 12 and 24 h, and the results suggest that AgNPs were able to reduce the cell viability of A2780 cells in a dose-dependent manner (Figure 2A). After 12 h of treatment, AgNPs were found to be cytotoxic to the cells at concentrations of 200 ng/mL but this effect was significant at 10,000 ng/mL. When the same cells were treated with 20–10,000 ng/mL for 24 h, significant cytotoxicity was observed even at 50 ng/mL (Figure 2B). It suggests that the effect of AgNPs is clearly influenced by the time of incubation and dose. Finally, we determined minimum inhibitory concentration of AgNPs at 24 h, which was found to be 1000 ng/mL (Figure 2B). Interestingly, at higher concentration at above 1000 ng/mL, the toxicity is not significant; it maintains the same level of toxicity.

### 2.3. Isolation and Characterization of Cancer Stem Cells (CSCs)

**Figure 3:**
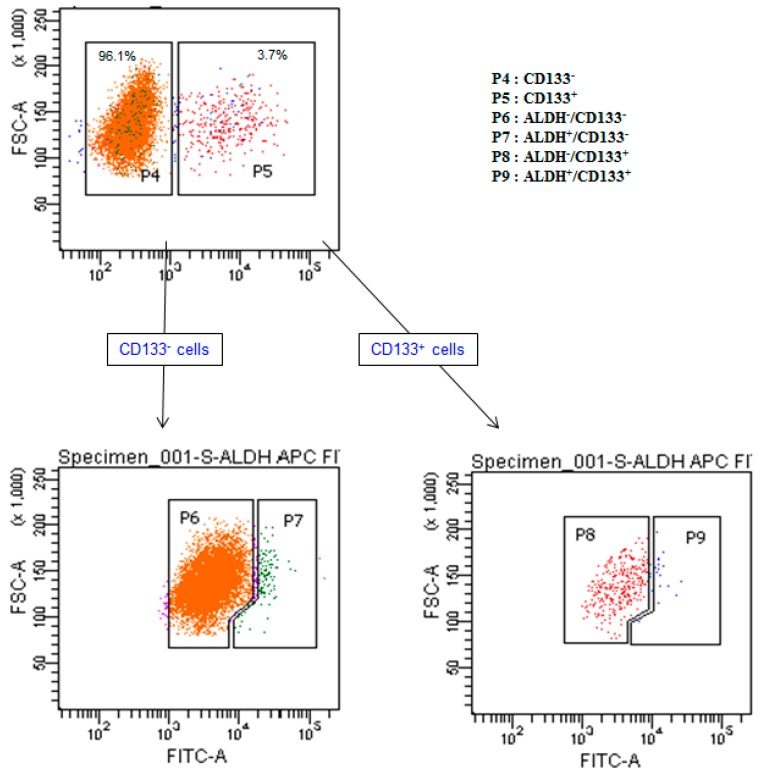
Schematic representation of expression of cancer stem cell (CSC) markers of ALDH and CD133 in human ovarian cancer cells using Fluorescent-activated cell sorting (FACS). P4: CD133^−^; P5: CD133^+^; P6: ALDH^−^/CD133^−^; P7: ALDH^+^/CD133^−^; P8: ALDH^−^/CD133^+^; P9: ALDH^+^/CD133^+^.

To determine the cytotoxic potential of AgNPs on different subpopulations of OvCSCs from A2780 cells, we first gated CD133 expression and then checked the expression of ALDH in CD133^−^ and CD133^+^ cell populations. The P9 gate resulted in the OvCSC population shown in Figure 3. To characterize the tumorigenic potential of different subpopulations of cells dually stained for ALDH expression and ALDH activity, ALDH^+^CD133^+^, ALDH^−^/CD133^+^, ALDH^+^/CD133^−^, and ALDH^−^/CD133^−^ cells were isolated from ovarian cancer cell lines. We characterized OvCSCs by the expression of potential CD133 and ALDH CSC markers, as these are characteristic markers for identification and isolation of CSCs from ovarian or other solid tumors [8,10,12]. ALDH was highly expressed and is the only potential stem cell marker expressed in all primary tumor specimens as well as limited cellular sub-populations of human primary tumor cells [10]. Hence, it could be a potentially useful CSCs marker in ovarian cancer. ALDH^+^/CD133^+^ cells could be used to increase the ability to generate tumor xenografts compared with ALDH^+^/CD133^−^ or ALDH^+^ alone [25]. ALDH^+^/CD133^+^ cells tend to elicit larger tumors and stimulate them more rapidly than ALDH^+^/CD133^−^ cells [25]. Based on literature and taking this into account, we scored different subpopulations including ALDH^+^/CD133^+^, ALDH^−^/CD133^+^, ALDH^+^/CD133^−^, and ALDH^−^/CD133^−^ cells from ovarian cancer cell lines and used them for further studies.

### 2.4. Effect of AgNPs on OvCSCs

**Figure 4:**
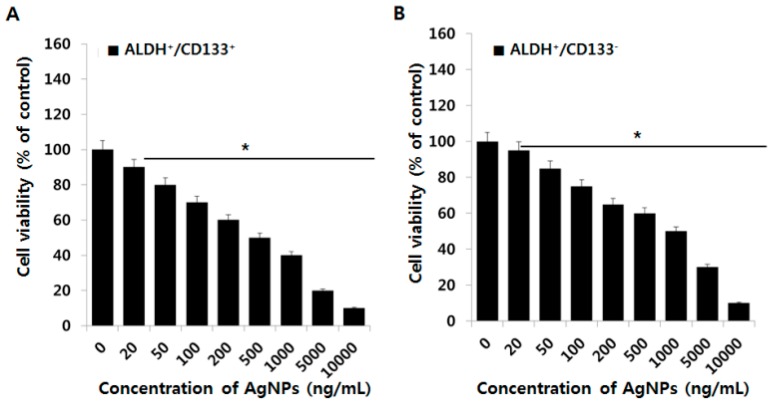

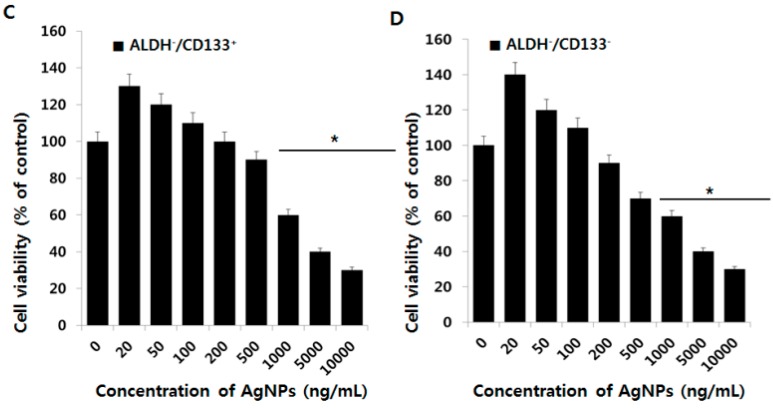
Effects of various concentration of AgNPs on cell viability of various subpopulations of OvCSCs. The viability of ALDH^+^/CD133^+^ (**A**) ALDH^−^/CD133^+^ (**B**) ALDH^+^/CD133^−^ (**C**) and ALDH^−^/CD133^−^ (**D**) cells was determined after 24-h exposure to AgNPs using the CCK-8 assay. The results are expressed as the mean ± standard deviation of three independent experiments. The viability of treated cells compared to the untreated cells was analyzed using the Student’s *t*-test (* *p* < 0.05).

CSCs are believed to occupy a limited percentage of solid tumors, and CSCs could be responsible for cancer relapses despite complete clinical remission with initial treatment. Many researchers are concentrating on the identification and development of new anticancer drugs with apoptosis-inducing properties, with a focus on CSCs. AgNPs are known to inhibit cancer cell viability in several cancer cell lines such as human breast, lung, and ovarian cancer cells [20,21,26]. Therefore, in this study we selected AgNPs as a potential alternative therapeutic agent for OvCSCs. AgNPs have a dual role: at lower concentrations, they can enhance cell survival and differentiation, and at higher concentrations, they can inhibit cell viability in neuronal cells [27]. For instance, AgNPs with an average size of 20 nm and at concentrations up to 2 µg/mL promoted osteogenic differentiation of urine-derived stem cells by inducing actin polymerization and activation of RhoA; whereas AgNO_3_ had no such effects [28]. To determine the effect of AgNPs on cell survival of four different subpopulations of OvCSCs, we first examined the dose-dependent effect of AgNPs. In order to assess the sensitivity or resistance of OvCSCs, the four populations of cells were incubated with different concentrations of AgNPs (20–10,000 ng/mL) for 24 h. As shown in Figure 4, dose-dependent inhibition of the cell viability was observed in each subpopulation of cells in the concentration range of 20–10,000 ng/mL with an IC_50_ (inhibitory concentration) value ranging from 1000–2000 ng/mL. The findings suggest that all four different subpopulations of cells exhibited enhanced cell viability after treatment with AgNPs concentrations at least up to 100 ng/mL except two subpopulations, such as ALDH^+^/CD133^+^ and ALDH^−^/CD133^+^, and a significant inhibitory effect was observed between 20 and 10,000 ng/mL, which depends on the subpopulation of cells. For example, ALDH^+^/CD133^+^ cells treated with AgNPs at concentrations from 20–200 ng/mL, there was a significant inhibition in cell viability was observed, and a dramatic effect was observed between 500 and 10,000 ng/mL, with an IC_50_ of ~1000 ng/mL (Figure 4A). When compared to bulk cells, it shows significant higher toxicity in dose dependent manner at above 1000 ng/mL. In ALDH^+^/CD133^−^ cells, a similar effect on cell viability was observed upon treatment with AgNPs at a concentration up to 200 ng/mL, and a significant inhibitory effect was observed at concentrations between 500 and 10,000 ng/mL, with an IC_50_ of ~1200 ng/mL (Figure 4B). In ALDH^−^/CD133^+^ cells, a dose-dependent inhibition in cell viability was observed in the AgNPs range of 500–10,000 ng/mL, with an IC_50_ of ~1500 ng/mL. However, at lower concentrations, there was no significant inhibitory effect. There was a positive effect on cell viability up to 500 ng/mL (Figure 4C). In ALDH^−^/CD133^−^ cells, a dose-dependent inhibition in cell viability was observed with AgNPs treatment in the range of 200–10,000 ng/mL, with an IC_50_ of ~1500 ng/mL (Figure 4D). ALDH^+^/CD133^+^ was shown to have more sensitivity to AgNPs, and a significant inhibitory effect on cell viability was observed compared with the other tested subpopulations of cells. Interestingly, ALDH^+^/CD133^+^ cells were more sensitive even at lower concentrations of AgNPs than the other tested subpopulations of cells. Altogether, the results suggest that ALDH^+^/CD133^+^ cells are a promising target cell type, to inhibit the viability of ovarian cancer stem cells. Generally, CSCs cell survival are governed by several signaling mechanism such as Notch, Hedgehog, Wnt, Her2, and IL-6 and -8 signaling pathways. Wnt signaling could be possible target for loss of viability in ALDH^+^/CD133^+^ cells. However the mechanism of sensitivity is not known. The differential IC_50_ value of each subpopulation of cells indicates that sensitivity of each cell type against AgNPs. Previous studies by Choi et al. [12] showed that only ALDH^+^/CD133^+^ cells could generate all four ALDH^+/−^CD133^+/−^ cell populations and identified a clear branched differentiation hierarchy. Therefore, further studies were focused only on the ALDH^+^/CD133^+^ subpopulation of cells.

### 2.5. Cytotoxic Effects of AgNPs on A2780 and OvCSCs

**Figure 5:**
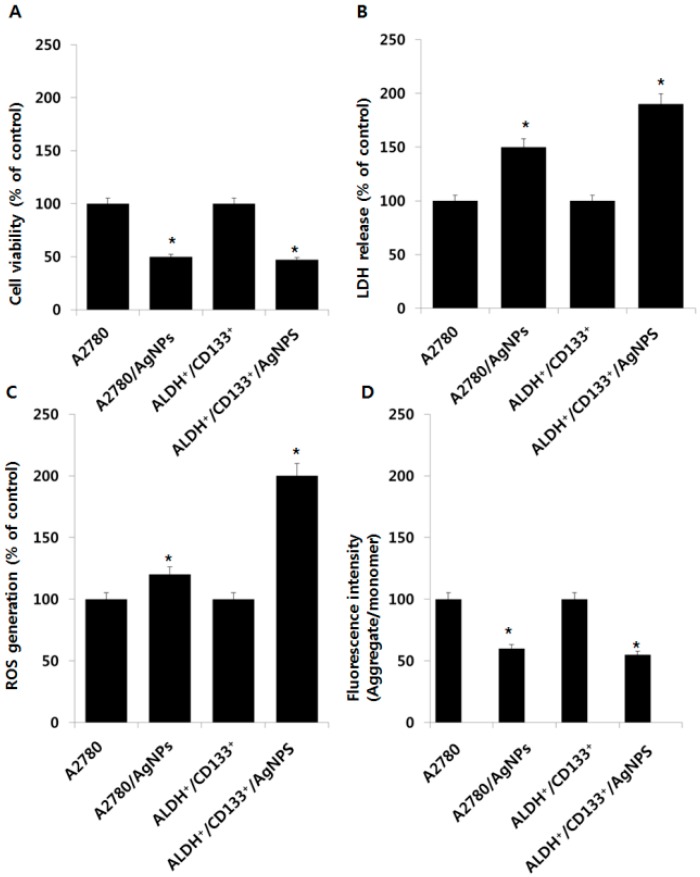
Effect of AgNPs on various cytotoxicity parameters in bulk cells and ALDH^+^/CD133^+^. Bulk cells and ALDH^+^/CD133^+^ were incubated with AgNPs (1000 ng/mL) for 24 h. Cell viability was determined using cell counting kit (CCK-8) assay (**A**); lactate dehydrogenase (LDH) activity was measured at 490 nm using the LDH cytotoxicity kit (**B**); reactive oxygen species (ROS) generation was determined by 2’,7’-dichlorofluorescein diacetate (DCFDA) (**C**); mitochondrial transmembrane potential (MTP) was determined using the cationic fluorescent indicator, JC-1 (**D**). The results are expressed as the mean ± standard deviation of three independent experiments. The treated groups showed statistically significant differences from the control group by Student’s *t*-test (* *p* < 0.05).

The above experimental conditions exhibited that low concentrations of AgNPs decrease the cell viability of ALDH^+^/CD133^+^ and ALDH^+^/CD133^−^ and increased the cell viability of ALDH^−^/CD133^+^ and ALDH^−^/CD133^−^ cells, therefore, we selected the respective IC_50_ value for each type of stem cell and tested the cytotoxic effects of AgNPs by assessing CCK-8, lactate dehydrogenase (LDH) release, reactive oxygen species (ROS) generation, and mt-MP (mitochondrial membrane potential). Particularly, we selected ALDH^+^/CD133^+^ cells, because it shows more sensitivity than other subpopulations. In the following experiment, we used A2780 cells as a positive control, which are parental of cells for all four different subpopulation of cells. Using their respective IC_50_ values for AgNPs, both bulk cells (A2780) and ALDH^+^/CD133^+^ cells were treated with AgNPs for 24 h, and the cell viability was examined (Figure 5A). When the A2780 and ALDH^+^/CD133^+^ cells were treated with AgNPs, there was a reduction in viability at IC_50_ concentrations of 1000 ng/mL in both parental cells as well as ALDH^+^/CD133^+^. An interesting observation in this experiment was that both bulk cells and ALDH^+^/CD133^+^ cells appeared equally sensitive to AgNPs, which indicated that AgNPs have significant cytotoxicity towards cancer stem cells and bulk cells. Anthothecol-encapsulated poly lactic-co-glycolic acid (PLGA)-nanoparticles inhibited cell proliferation and colony formation and induced apoptosis in pancreatic CSCs and cancer cell lines, but had no effect on human normal pancreatic epithelial cells [29].

LDH is a well-known marker for cell membrane integrity and cell viability, and its accumulation is the result of the breakdown of the plasma membrane and the alteration of its permeability at the stage of secondary necrosis at the late stage of apoptosis [30,31]. To assess the cytotoxic response to AgNPs, the amount of LDH leakage in the cell culture medium was measured at 24 h in bulk cells and ALDH^+^/CD133^+^ cells. Both bulk cells and ALDH^+^/CD133^+^ cells released LDH into the media (A2780) (Figure 5B). Among these two different types of cells, the ALDH^+^/CD133^+^ subpopulation showed greater sensitivity than bulk cells. Interestingly, the leakage of LDH in ALDH^+^/CD133^+^ cells was higher than in bulk cells (A2780). 

Next, we examined cytotoxic effects using the ROS generation assay. As expected, ALDH^+^/CD133^+^ cells produced higher amounts of ROS whereas bulk cells (A2780) produced less ROS (Figure 5C). The increase in ROS levels in cancer cells is partially due to their higher metabolism rate. Lower levels of ROS in bulk cells are due to the drug-resistant or chemoresistant CSCs population found in the bulk cells, which might use redox regulatory mechanisms to promote cell survival and tolerance to anticancer agents [32]. The possible reasons for lower levels of ROS in bulk cells are less ROS production, enhanced ROS scavenging systems, and the slow division of CSCs found in the bulk cells [33]. 

mt-MP reflects the functional status of the mitochondrion related to cancer malignancy [34]. Recent studies suggest that mitochondrial features are different in CSCs with respect to mt-MP and ROS [35,36]. To determine the mechanisms of ROS-mediated toxicity in bulk and OvCSCs, we assessed mt-MP. Several studies in cancer cells have shown that AgNP-induced ROS plays an important role in the formation of the mitochondrial permeability transition pore (MPTP), which eventually leads to activation of the mitochondria-dependent cell death pathways [21,37]. Thus far, there have been no reports on the comparative effects of AgNPs on the mt-MP in cancer cells or in OvCSCs. Therefore, we analyzed mt-MP using mitochondrial fluorescence dye, JC-1, which stains mitochondria in a membrane potential-dependent manner, in bulk cells and ALDH^+^/CD133^+^ cells treated with AgNPs. As shown in Figure 5D, bulk cells and ALDH^+^/CD133^+^ cells exposed to 1000 ng/mL AgNPs for 24 h exhibited a significant decrease in the ratio of aggregate to monomer forms. The results suggest that AgNPs have a significant impact on mt-MP in bulk cells as well as in ALDH^+^/CD133^+^ cells. The mt-MP is an indicator of the functional status of mitochondria, which is thought to correlate with a cell’s differentiation status, tumorigenicity, and malignancy [36]. The mitochondrial permeability is responsible for the release of apoptotic proteins such as cytochrome c and second mitochondria-derived activator of caspase (Smac), from the inter membrane space into the cytosol [38]. The functional status of mitochondria depends on mt-MP, which is highly related to cancer malignancy. Altogether, the data suggest that AgNPs could regulate the level of mt-MP and, in turn, induce apoptosis in both bulk cells and OvCSCs. Based on the cytotoxicity assays, ALDH^+^/CD133^+^ subpopulations seem to be more sensitive than bulk cells. Therefore, we selected ALDH^+^/CD133^+^ cells for further study. 

### 2.6. AgNPs Inhibit Colonies Formation

**Figure 6:**
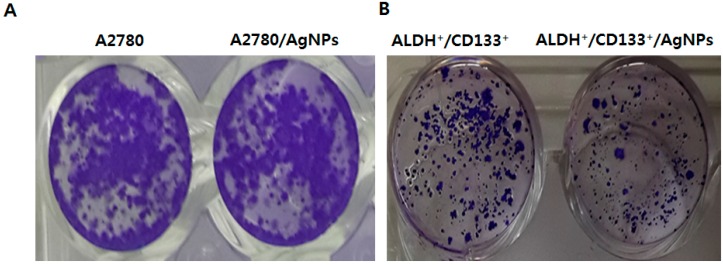

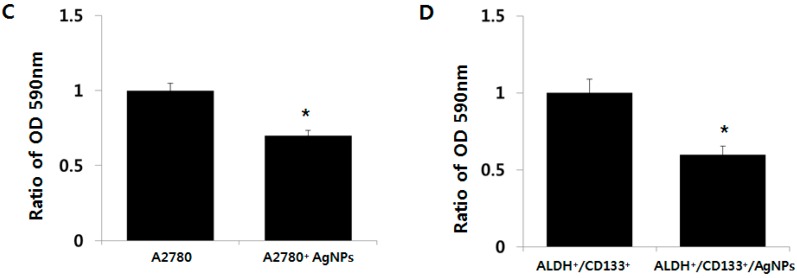
Effect of AgNPs on clonogenicity of A2780 (bulk cells) and ALDH^+^/CD133^+^. A2780 (**A**) and ALDH+/CD133+ (**B**) were seeded in RPMI-1640 with 10% fetal bovine serum (FBS) at a density of ~500 cells/well on 48-well plates that were pre-coated with Matrigel. After ~14 days, the colony formation ability was assessed by counting the number of colonies under a microscope after crystal violet staining. Representative images were photographed. For quantitative analysis of A2780 colony formation, crystal violet was completely dissolved in methanol and then the absorbance was measured at 590 nm (**C**); for quantitative analysis of ALDH^+^/CD133^+^ colony formation, crystal violet was completely dissolved in methanol and then the absorbance was measured at 590 nm (**D**). The results are expressed as the mean ± standard deviation of three independent experiments. The treated groups showed statistically significant differences from the control group by Student’s *t*-test (* *p* < 0.05).

To investigate whether AgNPs could impair the colony-formation capacity of A2780 and ALDH^+^/CD133^+^ cells, subpopulations were tested. After seeding the same number of both bulk and ALDH^+^/CD133^+^ subpopulations of cells, cells were cultured with AgNPs for ~14 days. After ~14 days, the colony formation ability was assessed by counting the number of colonies under a microscope after crystal violet staining. The results indicate that AgNPs-treated bulk A2780 cells had a significantly lower number of colonies compared to non-treated whole A2780 cells (Figure 6A). Similarly, ALDH^+^/CD133^+^ cells treated with AgNPs showed fewer numbers of colonies than untreated cells. When compared to bulk cells, ALDH^+^/CD133^+^ cells had a significant reduction in the number of colonies (Figure 6B). Altogether, the data from the cytotoxicity assays and clonogenecity assay showed that AgNPs were more cytotoxic to ALDH^+^/CD133^+^ OvCSCs than whole A2780 cells. To gain further evidence, we performed a quantitative analysis by dissolving crystal violet completely in menthol and then measuring absorbance at 590 nm. The relative absorbance showed the efficiency of AgNPs on inhibition of colony formation. The data demonstrated that AgNPs treatment of A2780 cells is an effective method for reducing the OvCSCs population in heterozygote ovarian tumors. Particularly, specific targeting of ALDH^+^/CD133^+^ cells by AgNPs is a suitable, efficient, and alternative method for cancer therapy. However, the mechanism of sensitivity of ALDH^+^/CD133^+^ cells are not known.

### 2.7. AgNPs Induce Differential Apoptotic Responses in Bulk Cells (A2780) and OvCSCs (ALDH^+^/CD133^+^)

**Figure 7:**
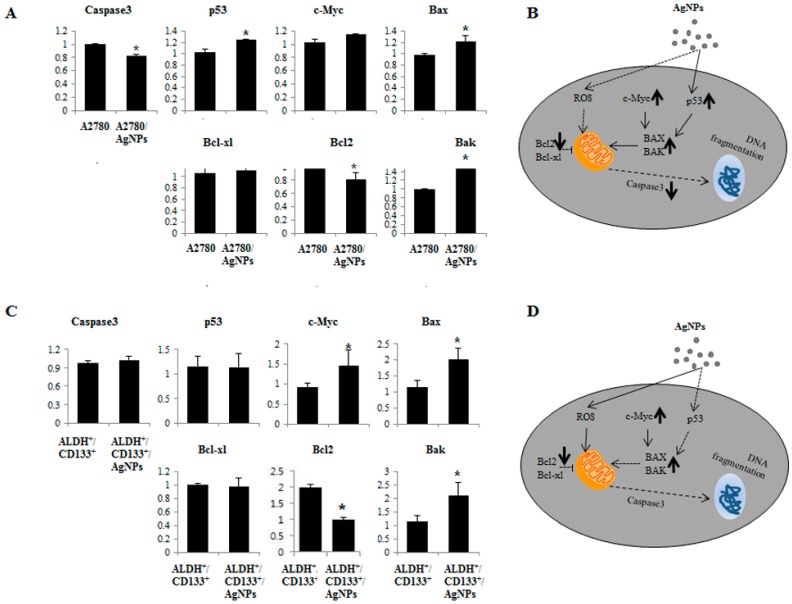
Effect of AgNPs on expression of apoptosis-regulated genes in bulk cells and ALDH^+^/CD133^+^. Relative mRNA expression of various apoptotic genes were analyzed by qRT-PCR in A2780 cells and ALDH^+^/CD133^+^. A 2780 cells were treated with AgNPs (1000 ng/mL) for 24 h and expression was analyzed (**A**). The schematic representation of mechanism of cell death of A2780 cells were illustrated (**B**); ALDH^+^/CD133^+^ cells were treated with AgNPs (1000 ng/mL) for 24 h and expression was analyzed (**C**); The schematic representation of mechanism of cell death of ALDH^+^/CD133^+^ cells were illustrated (**D**). The results are expressed as the mean ± standard deviation of three separate experiments. The treated groups showed statistically significant differences from the control group by Student’s *t*-test (* *p* < 0.05). Schematic representation of AgNPs induced apoptosis by up-regulation or down-regulation apoptotic related proteins (The symbols bold arrow indicate high expression of genes; solid arrow means indicate moderate expression of genes; dotted arrow indicate that lower expression of genes; T-bar indicate inhibition).

The cell viability, cytotoxicity, and colony formation assays suggested that OvCSCs were more sensitive to AgNPs than the A2780 cells at respective IC_50_ concentrations of AgNPs. Based on these outcomes, it was favorable to use A2780 and ALDH^+^/CD133^+^ cells to explain the mechanism of apoptosis. To address this issue, apoptotic gene expression analysis was performed to examine pro-apoptotic genes (*p53*, *caspase 3*, *bax*, *bak*, and *c-myc*) and anti-apoptotic genes (*bcl-2* and *bcl-xl*) using real-time reverse transcription polymerase chain reaction (RT-PCR) in both bulk cells and ALDH^+^/CD133^+^ cells exposed to AgNPs for 24 h. The results indicated up-regulation of *p53*, *bax*, *bak*, and *c-myc* genes (indicated by upward arrow in Figure 7B) and down-regulation of *bcl-2* (indicated by downward arrow in Figure 7B) in AgNPs treated A2780 cells compared with untreated A2780 cells (Figure 7A,B). The expression of β-actin remained the same. Overall, the sequence of events leading to apoptosis in AgNPs-treated A2780 cells is illustrated in Figure 7B. The AgNPs could induce oxidative stress in A2780 cells by generating higher levels of ROS and triggering the p53-mediated apoptotic pathway, whereas the later event of apoptosis carried out by caspase-3 has no impact on bulk cells. In case of ALDH^+^/CD133^+^, AgNPs treated cells shows up-regulation of caspase-3, *bax*, *bak*, and *c-myc*, genes (indicated by upward arrow in Figure 7D) was observed in AgNPs treated ALDH^+^/CD133^+^ compared with untreated ALDH^+^/CD133^+^ (Figure 7C,D). Interestingly, there is no significant effect on p53 and Bcl-xl in AgNPs treated ALDH^+^/CD133^+^. This also indicates that AgNPs regulate apoptosis in a differential manner in bulk cells and ALDH^+^/CD133^+^. Overall, the sequence of events leading to apoptosis in both AgNPs-treated A2780 and ALDH^+^/CD133^+^ cells were shown in Figure 7A–D. The data suggest that AgNPs induces apoptosis by oxidative stress, in which Bcl-2 playing an important role in mitochondrial outer membrane permeabilization and loss of mitochondrial membrane potential.

The process of apoptosis is positively regulated by the tumor-suppressor p53, which induces the expression of many pro-apoptotic genes, including death receptors and multiple pro-apoptotic Bcl-2 family members [39]. p53 suppresses proliferation and self-renewal of neural stem cells [40]. The Bcl-2 family proteins play a pivotal role in mitochondrial-mediated apoptosis [41]. The anti-apoptotic proteins prevented cytochrome c release by forming heterodimer complexes with pro-apoptotic Bcl-2 family proteins, and Bax facilitates the release of apoptogenic molecules from mitochondria to the cytosol and accelerates apoptotic cell death [42,43,44]. The Bcl-2 protein family plays an integral role in maintaining the balance between cell survival and apoptosis. Based on our findings, the possible mechanism of inhibition of Bcl-2 by AgNPs could be the induction of mitochondrial dysfunction and energy depletion in CSCs in turn inducing the imbalance between oxidant and antioxidant levels in the cells. Inhibition of Bcl-2 and Bcl-xl by ABT-737 in tyrosine kinase inhibitor (TKI)-resistant blast crisis (BC) chronic myeloid leukemia (CML) promotes apoptosis in quiescent CD34^+^ CML stem cells [45]. In addition to suppressing anti-apoptotic Bcl-2 family members, activation of pro-apoptotic Bax is required for activation of apoptosis through the mitochondria. The berberine liposome induces apoptosis via down-regulation of *Bcl-2* and up-regulation of *Bax* in colon CSCs [46]. The loss of mt-MP might promote activation of cytochrome c and mitochondria-derived caspases. The results from our experiment suggest that AgNPs can up-regulate the expression of p53 and caspase-3 in bulk ells and ALDH^+^/CD133^+^ subpopulations, respectively. For instance, 20 (s)-ginsenoside Rg3 inhibits proliferation of colon CSCs and induces apoptosis through caspase-9 and caspase-3 pathways.

## 3. Materials and Methods

Penicillin-streptomycin solution, trypsin-Ethylenediaminetetraacetic acid (EDTA) solution, Dulbecco’s modified Eagle’s medium (DMEM), Roswell Park Memorial Institute (RPMI) 1640 medium, and 1% antibiotic-antimycotic solution were obtained from Life Technologies/Gibco (Grand Island, NY, USA). Fetal bovine serum (FBS) and the in vitro toxicology assay kit were purchased from Sigma-Aldrich (St. Louis, MO, USA).

### 3.1. AgNPs Characterization

AgNPs were obtained from Nano High Tech (Seoul, Korea) as a clear colloidal aqueous suspension with a concentration of 1mg/mL. AgNPs characterization was performed as previously described [24]. AgNPs were primarily characterized by UV-VIS spectroscopy. Ultraviolet-visible (UV-VIS) spectra of AgNPs were recorded using an OPTIZEN POP spectrophotometer (Mechasys, Seoul, Korea) and other characterization was performed as described previously [24].

### 3.2. Cell Culture and Exposure to AgNPs

A2780 cell lines were kindly provided by Prof. Ronald Buckanovich, Division of Gynecologic Oncology, Department of Obstetrics and Gynecology, University of Michigan Medical Center, Ann Arbor, MI, USA. The cells were cultured in RPMI-1640 supplemented with 10% fetal bovine serum (FBS), 100 U/mL penicillin, and 100 µg/mL streptomycin. Attached cells were fully disaggregated by trypsinization between passages. The culture medium was replaced with medium containing AgNPs prepared at specific concentrations (0–10,000 ng/mL). After incubation for an additional 24 h, the cells were collected and analyzed for cell viability and other cytotoxicity assays. The cell lines were maintained at 37 °C in an incubator with humidified air with 5% CO_2_.

### 3.3. Flow Cytometry Analysis and Fluorescence-Activated Cell Sorting (FACS)

FACS was performed according to the method described previously [10] with suitable modifications. Cell line single-cell suspensions were counted and incubated with CD133 primary antibodies, and then ALDH^+^ enzymatic activity was defined using the ALDEFLUOR kit per the protocol (Stem Cell Technologies, Vancouver, BC, Canada). For each sample, half of the cell/substrate mixture was treated with 50 mmol/L diethylaminobenzaldehyde (DEAB). Cells were incubated for 45 min. Gating for viability was established using propidium iodide (PI) exclusion and ALDEFLUOR/DEAB-treated cells were used to define negative gates. FACS was performed with ≥1 × 10^5^ cells using the BD FACSCanto II (Becton Dickinson, Franklin Lakes, NJ, USA) or FACSAria (Becton Dickinson) under low pressure in the absence of UV light. In all experiments, the ALDEFLUOR-stained cells treated with DEAB served as ALDH-negative controls. The ALDH^+^CD133^+^, ALDH^−^CD133^+^, ALDH^+^CD133^−^, and ALDH^−^CD133^−^ subpopulations were separated from the A2780 ovarian cancer cells by a FACSAria (Becton Dickinson). After sorting, all the cell subpopulations were cultured in a RPMI-1640 basic culture medium for 2 h; then, the cells were treated with different nanomaterials such as GO (50 μg/mL), rGO (20 μg/mL), rGO-Ag nanocomposite (10 μg/mL), and AgNPs (15 μg/mL) for 24 h.

### 3.4. Cell Viability (CCK-8 Assay)

The CCK-8 assay was analyzed according to the method described previously [26,31]. The cells were seeded in a 96-well plate and cultured in DMEM supplemented with 10% FBS for 24 h and incubated with various c concentrations of AgNPs for 24 h. 

### 3.5. Reactive Oxygen Species Assay (DCFH-DA)

DCF-DA was performed according to the manufacturer protocol. A2780 human ovarian cancer cells and sorted cells were cultured in RPMI-1640 medium containing 10 μM H_2_-DCFDA in a humidified incubator at 37 °C for 30 min. Cells were washed in phosphate buffered saline (PBS), and tested at an excitation wavelength of 480 nm and emission wavelength of 530 nm using a GeminiEM fluorescence multiplate reader (Molecular Devices, Sunnyvale, CA, USA).

### 3.6. Mitochondrial Transmembrane Potential Assay (JC-1)

The mt-MP assay was performed following the manufacturer protocol. The cells were treated with 1000 ng/mL of AgNPs and then mt-MP was measured using the cationic fluorescent indicator, JC-1 (Molecular Probes Eugene, OR, USA). 

### 3.7. Clonogenic Assay 

The clonogenic assay was performed as previously described with modifications [47]. A2780 whole cells and sorted cells were plated on 48-well plates at a density of 100 cells per well and were allowed to adhere for 18 h. The cells were incubated with AgNPs 1000 ng/mL were added to each well and incubated for a maximum of 14 days at 37 °C. At least 50 cells were counted from each colony by manually. All data are expressed as relative to control.

### 3.8. RT-PCR Analysis

Total RNA was extracted from cells treated with AgNPs using an Arcturus PicoPure RNA isolation kit (ABioscience, San Diego, CA, USA) according to the manufacturer’s instructions. RNA was reverse transcribed into cDNA using a Reverse Transcription Kit (Roche) in a final volume of 20 μL according to manufacturer instructions. The quantification of all gene transcripts (*caspase3*, *p53*, *c-myc*, *Bax*, *Bad*, *Bcl-xl*, *Bcl2*, and *Bax*) was carried out in three replicates by real-time reverse transcriptase quantitative polymerase chain reaction (RT-qPCR) on a Lightcycler apparatus using Lightcycler^®^FastStart DNA Master SYBR Green I via an ABI Applied Biosystems machine. The primer sequences for each gene are shown in Table 1. The relative gene expression was quantified and analyzed by the 2^−∆∆*C*t^ method. In all experiments, *GAPDH* mRNA was used as an internal standard.

### 4. Conclusions

Sequential self-renewal and the differentiation of cancer stem cells are responsible for tumor recurrence after treatment with radiation or chemotherapy and current therapies fail to eliminate CSCs. The cytotoxic effect of AgNPs on ovarian cancer stem cells is an unexplored area, which could shed light on the mechanisms of toxicity as well as on potential therapeutic agents. In the present study, we selected A2780 cells as model for isolating CSCs. Since A2780 is known to express aldehyde dehydrogenase (ALDH) activity, which is reported CSC marker in several solid tumors including ovarian cancer, and also the small number of ALDH^+^ cells are capable of tumor initiation and propagation, and these cells generate tumors which recapitulate the original tumor cell composition. In addition to these, A2780 cell lines are poorly differentiated, highly tumorigenic, and het­erogeneous with certain phenotypic subsets attributable to CSC-like properties when compared other ovarian cancer cell lines. Furthermore, these cells demonstrate resistance to chemotherapy and increased angiogenic capacity [10]. Choi et al. [12] reported that ALDH^+^/CD133^+^ subpopulation of cells could generate all four ALDH^+/−^CD133^+/−^ cell populations and had a clear branched differentiation hierarchy. Therefore we selected A2780 ovarian cancer cells to investigate the effect of AgNPs in both bulk cells (A2780) and CSCs derived from A2780 cells. The viability assays suggest that the cells treated with AgNPs showed different responses in A2780 ovarian cancer cells and four different subpopulation of CSCs derived from A2780 cells. Furthermore, cytotoxicity assays clearly indicated ALDH^+^/CD133^+^ more sensitive than bulk cells. The evidence gained from cytotoxicity assays supports that the interaction of nanoparticles with the cell membrane triggered ROS generation and oxidative stress alterations in metabolic pathways and apoptosis. Treatment of cells with AgNPs had a significant effect on cell viability, LDH leakage, ROS generation, and loss of mt-MP. However, of these two cell types, OvCSCs seem to be more sensitive than bulk cells. Further evidence shows that AgNPs inhibited colony formation both in bulk and OvCSCs, and a severe effect was observed in OvCSCs. The results indicate that AgNPs can be used to specifically target ALDH^+^/CD133^+^ cells, providing a possible approach for cancer therapy without side effects. This is the first study providing evidence for the specific targeting of the ALDH^+^/CD133^+^ subpopulation of CSCs by AgNPs and differential regulation of AgNPs in bulk cells and ALDH^+^CD133^+^ cells. However, the mechanism of sensitivity of ALDH^+^/CD133^+^ is still unknown. Further studies are warranted and should focus on CSCs-specific detailed signaling pathways, surface markers, and mechanisms of apoptosis.

## Figures and Tables

**Table 1:** List of primers used in this study.

Gene	Primers
*Bcl2*	F: ATGTGTGTGGAGAGCGTCAA
	R: GCCGGTTCAGGTACTCAGTC
*c-myc*	F: AGCGACTCTGAGGAGGAACA
	R: CTCTGACCTTTTGCCAGGAG
*p53*	F: TTTGGGTCTTTGAACCCTTG
	R: CCACAACAAAACACCAGTGC
*Bax*	F: ATGGAGCTGCAGAGGATGAT
	R: CAGTTGAAGTTGCCGTCAGA
*Caspase-3*	F: CATACTCCACAGCACCTGGTTA
	R: ACTCAAATTCTGTTGCCACCTT
*Bcl-xL*	F: GTAAACTGGGGTCGCATTGT
	R: CGATCCGACTCACCAATACC
*Bak*	F: CTCAGAGTTCCAGACCATGTTG
	R: CATGCTGGTAGACGTGTAGGG
